# QT-related adverse events with ondansetron and olanzapine: a real-world FAERS analysis with implications for oncology anti-emetic practice

**DOI:** 10.3389/fphar.2026.1748635

**Published:** 2026-02-20

**Authors:** Ayush Gandhi, Alireza Parhizgar, Viraj Bhise

**Affiliations:** 1 Department of Hospital Medicine, The University of Texas MD Anderson Cancer Center, Houston, TX, United States; 2 Department of Emergency Medicine, The University of Texas MD Anderson Cancer Center, Houston, TX, United States

**Keywords:** FAERS, olanzapine, oncology, ondansetron, pharmacovigilance, QT prolongation, torsades de pointes

## Abstract

**Purpose:**

QT prolongation remains an important safety concern for antiemetic regimens, especially in oncology where polypharmacy and metabolic stress often intersect. Ondansetron and olanzapine are now used side by side in many chemotherapy and perioperative settings, yet their comparative real-world torsadogenic patterns are not fully understood. We aimed to evaluate the relative frequency of QT-related adverse event reports for both drugs using the FDA Adverse Event Reporting System (FAERS).

**Patients and Methods:**

We performed a retrospective pharmacovigilance study using OpenVigil 2.1 to extract adult FAERS reports from January 2015 through October 2025. Only primary suspect reports were included. Narrow-term events were defined using two specific MedDRA Preferred Terms: torsades de pointes and electrocardiogram QT prolonged. A broader ventricular arrhythmia set was used for sensitivity analysis. Standard disproportionality statistics (ROR, PRR, chi-square) were calculated, and subgroup analyses examined age, sex, and FAERS serious outcomes.

**Results:**

The analysis included 2,608 ondansetron reports and 15,257 olanzapine reports. Narrow-term QT events were more frequently reported with ondansetron (4.91 percent) than olanzapine (1.61 percent). Ondansetron showed a stronger disproportionality signal with an ROR of 27.24 (95 percent CI 22.78–32.58), compared with olanzapine’s ROR of 8.70 (95 percent CI 7.65–9.88). This pattern persisted, although at lower magnitudes, with the broader arrhythmia definitions. Female predominance was observed across both drugs, and most reports came from adults younger than 65 years. Hospitalization and life-threatening events were the most common serious categories.

**Conclusion:**

In real-world reporting, ondansetron demonstrates a descriptively higher QT-related disproportionality signal than olanzapine, a finding that aligns with their known electrophysiologic profiles and prior regulatory experience. Although olanzapine’s average QT effect is modest, its signal becomes more visible when clinical conditions amplify vulnerability, which is common in oncology. These results do not establish causality, yet they offer useful context when selecting antiemetic strategies for patients already carrying QT-related risks. Integrating pharmacovigilance patterns with clinical judgment may help clinicians tailor safer antiemetic choices, particularly when multiple QT-active agents are used together.

## Introduction

Chemotherapy-induced and postoperative nausea remain some of the most common symptoms clinicians try to pre-empt, and antiemetic prophylaxis has slowly become a multi-agent exercise. Ondansetron, a selective 5-HT_3_ antagonist, has been the long standing mainstay antiemetic in both oncology and perioperative practice for years, largely because it performs reliably and carries fewer legacy toxicities than older agents (Rezaei Zadeh Rukerd et al., 2024). Olanzapine entered this space much later. Although introduced as an atypical antipsychotic, its broad neurotransmitter antagonism turned out to be unexpectedly useful for refractory nausea. Several trials demonstrated that adding olanzapine (usually 5–10 mg) to standard regimens markedly improves control of chemotherapy-induced nausea and vomiting (CINV) (Zhang et al., 2025; Uchino and Shibutani, 2025). Current guidelines now treat it as a first-line component for highly emetogenic chemotherapy, which has rapidly normalized its use in oncology clinics (Bowen et al., 2025). In contrast, perioperative use of olanzapine remains more selective—an option considered for persistent nausea or when clinicians anticipate high-risk physiology (Grigio et al., 2023).

As these two drugs increasingly coexist in real clinical scenarios, especially in oncology patients who may receive both during a single cycle, the question of their comparative cardiac safety has become difficult to ignore. Each affects cardiac repolarization to some degree. QT interval prolongation, the electrophysiologic precursor clinicians worry about, remains the strongest mechanistic signal linked to torsades de pointes (TdP) (Ostroff, 2025; Tan et al., 2024). Ondansetron’s relationship with QT prolongation has been recognized for more than a decade; a now-removed 32 mg IV dose caused meaningful QTc prolongation and led to an FDA warning (U. S. Food and Drug Administration, 2021). Even the smaller, routinely used 4–8 mg IV doses can lengthen the QTc modestly, with the 8 mg range producing more noticeable changes (Rezaei Zadeh Rukerd et al., 2024). The mechanistic link blockade of the hERG (Human Ether-a-go-go-related gene) is well established (Ostroff, 2025).

Olanzapine, at standard antipsychotic or antiemetic doses, exerts a much subtler effect. Comparative antipsychotic studies consistently report only minimal mean QTc changes (about 1–3 msec) (Huhn et al., 2019; Chung and Chua, 2011), and the drug is rarely highlighted as a major QT-prolonging agent when used alone (Hasnain and Vieweg, 2014). However, its cardiac profile is not completely benign. Olanzapine also has hERG-blocking properties, and case reports continue to surface. Olanzapine is classified by CredibleMeds as a “possible” TdP risk agent, with risk primarily in the presence of triggers such as electrolyte disturbances or concomitant QT-prolonging drugs, as supported by the American Heart Association and ADECA (Adverse Drug Event Causality Analysis) process descriptions (Page et al., 2016; Woosley et al., 2017).

In oncology populations, the picture can be even more complicated. One prospective study examining olanzapine with ondansetron and domperidone found significant QTc increases over baseline among female patients undergoing chemotherapy (Kamath et al., 2021). These isolated observations remind clinicians that olanzapine’s generally small average effect can amplify once it enters a milieu crowded with other QT-active drugs.

This makes QT monitoring a central concern. Although serious arrhythmias remain uncommon, the consequences can be catastrophic (Al-Khatib et al., 2003; Tisdale et al., 2020; Fradley et al., 2021). Unfortunately, the pre-marketing trials that clinicians often rely on are rarely powered to detect rare ventricular arrhythmias, which pushes much of this learning into the post-marketing space. The FDA Adverse Event Reporting System (FAERS) has gradually become indispensable for this reason. It allows researchers to identify disproportionate clusters of drug-associated adverse events including TdP that may not be obvious in smaller controlled trials (Tan et al., 2024). Mining FAERS for comparative purposes, especially when two drugs occupy similar clinical niches, offers a practical way to understand how often clinicians are encountering real-world cardiac issues with each agent (Xu et al., 2024; Tang et al., 2022). For oncologists, anesthesiologists, and hospitalists facing daily antiemetic decisions, that kind of information has immediate value.

### Rationale and aim

Against this background, the present study uses FAERS data to compare cardiac safety signals associated with ondansetron and olanzapine in adults. We focus specifically on TdP and related serious ventricular arrhythmias. Our aim is not to draw causal conclusions but to examine whether one drug generates a consistently higher disproportionality signal than the other, and to interpret those patterns in light of their known electrophysiologic and clinical profiles. The hope is that these findings can help clinicians make more informed, risk-aware choices around antiemetic prophylaxis, particularly in patients with overlapping cardiac vulnerabilities. By integrating large-scale pharmacovigilance analysis with established mechanistic understanding, this work illustrates how real-world data can be translated into practical, safety-oriented decision-making.

## Materials and methods

### Data source and study design

We performed a retrospective pharmacovigilance study using OpenVigil 2.1, which retrieves, deduplicates, and structures data directly from the FDA Adverse Event Reporting System (FAERS). The analytic period spanned 1 January 2015 through 31 October 2025. Reports were included if they listed ondansetron or olanzapine as the primary suspect (PS) drug. FAERS drug roles designate the medication considered most likely to have contributed to the event (primary suspect), secondary suspect, concomitant, or interacting; only primary suspect reports were retained to enhance causality plausibility. Only adult patients (≥18 years) were included, and both U.S. and international reports were eligible.

### Sensitivity analysis by drug role

To assess the robustness of findings to case definition, we conducted a sensitivity analysis including reports where the drug of interest was listed as either primary suspect (PS) or secondary suspect (SS). This broader definition may capture events where the reporter was uncertain about attribution or where multiple drugs contributed to the adverse event. Disproportionality metrics (ROR, PRR, chi-square) were calculated for the PS + SS cohort using the same methodology as the primary analysis.

### FAERS serious outcome definitions

FAERS uses FDA MedWatch standardized categories to classify serious clinical outcomes. These include: death, life-threatening events, and hospitalization (initial or prolonged). These classifications originate from reporter submitted MedWatch forms and are incorporated into FAERS without modification. For this study, all serious outcomes were extracted exactly as defined by FAERS.

### Adverse event definitions and search strategy

The primary analysis focused on a narrow and specific set of MedDRA Preferred Terms (PTs) representing well-characterized drug-induced repolarization abnormalities. These included:

- Torsades de pointes (TdP).
- Electrocardiogram QT prolonged.

These PTs were chosen a priori, as they represent the most clinically specific electrophysiologic events linked to pharmacologic QT prolongation.

A secondary sensitivity analysis used a broader arrhythmia definition to detect more diffuse ventricular instability. This extended set included the narrow-term PTs plus: ventricular tachycardia, ventricular fibrillation, ventricular arrhythmia, ventricular tachyarrhythmia, long QT syndrome, cardiac arrest, and sudden cardiac death. All PT selections aligned with the MedDRA version embedded in OpenVigil 2.1 at the time of extraction.

### Quantification of exclusions missingness, data cleaning, and deduplication

We documented the number of reports excluded at each filtering step, including those excluded for missing age, missing primary suspect designation, and pediatric population. Exclusion counts and proportions were calculated separately for ondansetron and olanzapine to assess whether exclusions were differential between drugs. These data are presented in Supplementary Table S1.

OpenVigil removes duplicate reports by retaining the most recent case version using the FAERS ISR (Individual Safety Report) hierarchy. For each drug, we extracted total adverse event counts, narrow-term QT-event counts, demographics, and serious clinical outcomes when available.

### Subgroup analyses

Within the narrow-term cohort, additional subgroup analyses were conducted. Age was stratified into 18–64 years, 65–85 years, and older than 85 years. Sex distribution was evaluated when reported. Serious outcomes were tabulated using standard FAERS categories that include death, hospitalization, and life-threatening events.

### Stratified and sensitivity analyses for confounding

To address potential confounding and assess signal robustness, we conducted additional stratified analyses:

#### Calendar year stratification

To assess temporal stability of QT-related disproportionality signals and to explore potential effects of evolving prescribing patterns or regulatory communications, reports were stratified into three calendar periods: early (2015–2018), intermediate (2019–2021), and recent (2022–October 2025) (Supplementary Table S3).

#### Geographic subanalysis

To evaluate the robustness of findings within a more homogeneous reporting environment, a prespecified subanalysis was performed restricting the dataset to U.S. reports. Results from this U.S.-only analysis were compared descriptively with the primary analysis, which included both U.S. and international FAERS reports (Supplementary Table S4).

### Construction of 2 × 2 contingency tables

For each drug, standard 2 × 2 contingency tables were constructed for disproportionality analysis:

**Table:** 

​	QT-related event (case of interest)	All other events	Total
Drug of interest	a	b	a + b
All other drugs	c	d	c + d
Total	a + c	b + d	N

Where a represents event reports for the drug, b represents other reports for the drug, c represents event reports for all other drugs, and d represents non-event reports for all other drugs.

### Statistical interpretation of between-drug comparisons

This analysis was intended to identify safety signals rather than to perform a formal head-to-head comparative hypothesis test. Disproportionality metrics were therefore estimated separately for each drug using the full FAERS database as the reference. Although the absence of overlap between confidence intervals can suggest differences in signal magnitude, this should be viewed as indirect evidence only and not as a definitive between-drug comparison. In spontaneous reporting systems, formal comparative inference is constrained by the lack of exposure denominators, variability in reporting behavior, and residual confounding related to clinical context and indication. For these reasons, the present findings are best interpreted as descriptive and hypothesis generating, serving to highlight relative reporting patterns rather than to establish comparative risk.

### Disproportionality analysis: ROR, PRR and chi-square

Signal detection was performed using Reporting Odds Ratio (ROR) and Proportional Reporting Ratio (PRR), the two primary frequentist methods recommended in pharmacovigilance.

#### Reporting odds ratio (ROR)

The ROR assesses the odds of reporting a QT-related event with the drug *versus* all other drugs. It was calculated as:

$$\text{ROR}=\left(a*d\right)\;/\;\left(b*c\right)$$

Ninety-five percent confidence intervals were automatically generated using OpenVigil’s log-transformed variance method. A signal was considered present when the lower bound of the 95% CI exceeded 1.0.

#### Proportional reporting ratio (PRR)

The PRR measures the proportion of QT-related events among all reports involving the drug relative to the same proportion for all other drugs.

$$\text{PRR}=\left(a/\left(a+b\right)\right)\;/\;\left(c/\left(c+d\right)\right)$$

Statistical significance of PRR was assessed using the chi-square statistic, calculated as:

$$\chi^{2}=\left({\left(a^{*}d-b^{*}c\right)}^{2}*\left(a+b+c+d\right)\right)/\left(\left(a+b\right)\left(c+d\right)\left(\;a+c\right)\left(b+d\right)\right)$$

A signal was considered present per Evans criteria (Evans et al., 2001):

- PRR ≥2,
- at least three cases (a ≥3), and
- chi-square ≥4.

Confidence intervals were calculated by OpenVigil 2.1 using standard log-scale variance formulas for disproportionality metrics, as outlined in the OpenVigil statistical documentation.

**FIGURE 1:**
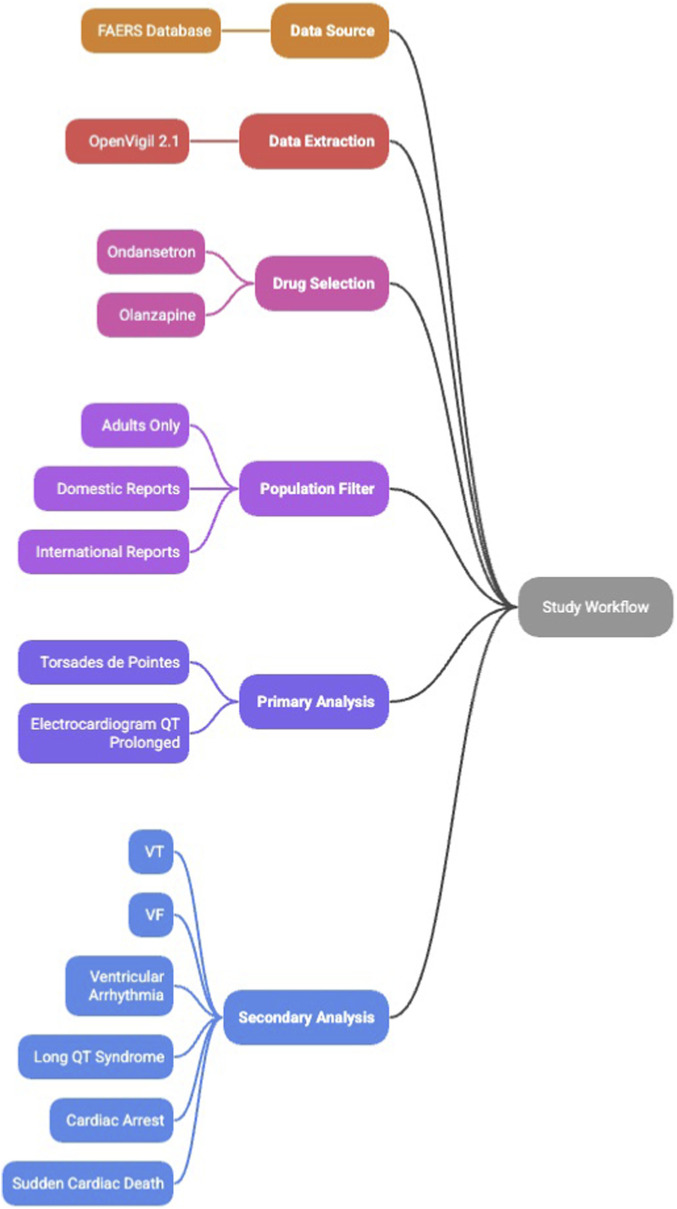
Pharmacovigilance study workflow.

Workflow summarizing the pharmacovigilance analysis using FAERS data extracted through OpenVigil 2.1. Reports were limited to adults older than 18 years and included both domestic and international submissions. Ondansetron and olanzapine were analyzed as primary suspect drugs. The primary analysis used narrow MedDRA Preferred Terms (torsades de pointes and electrocardiogram QT prolonged). A secondary analysis incorporated broader ventricular arrhythmia terms, including ventricular tachycardia, ventricular fibrillation, ventricular arrhythmia, long QT syndrome, cardiac arrest, and sudden cardiac death. Subgroup analyses evaluated age categories, gender, and serious clinical outcomes. Disproportionality metrics (ROR, PRR, Chi Square) were calculated for each drug-event pair. The overall study workflow is summarized in Figure 1.

#### Ethical considerations

The study used de-identified, publicly available FAERS data. Institutional Review Board approval and informed consent were not required.

## Results

### Overall dataset

**FIGURE 2:**
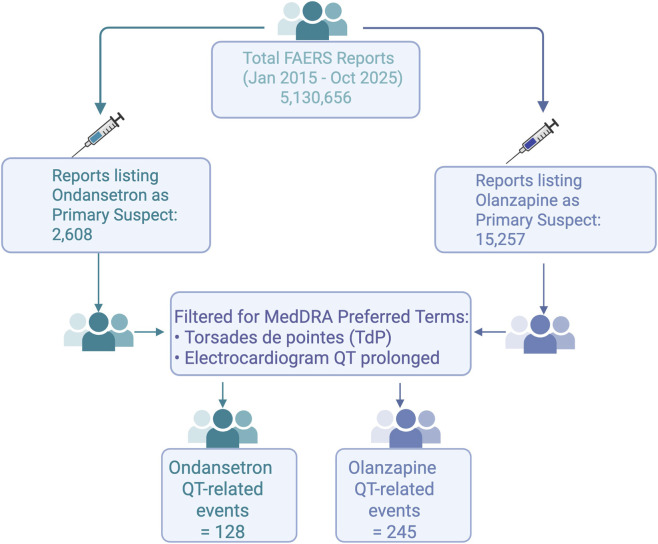
FAERS data selection process.

From 1 January 2015 through 31 October 2025, a total of 2,608 ondansetron reports and 15,257 olanzapine reports met inclusion criteria after filtering for adult age, primary suspect role, and deduplication (Figure 2). Narrow-term QT-related events were identified using the MedDRA Preferred Terms torsades de pointes and electrocardiogram QT prolonged. Broader ventricular arrhythmia terms were used for the secondary analysis. Figure 2 demonstrates the data selection process.

### Exclusion flow and missingness

From the initial FAERS query, 16,092 ondansetron reports and 49,169 olanzapine reports were identified. After applying inclusion criteria, 83.8 percent of ondansetron reports and 69.0 percent of olanzapine reports were excluded. The primary reasons for exclusion were missing age information (ondansetron 23.3 percent, olanzapine 27.7 percent), non–primary suspect designation (ondansetron 52.2 percent, olanzapine 36.1 percent), and pediatric age (ondansetron 8.3 percent, olanzapine 5.2 percent). Exclusion rates were somewhat differential between drugs, which may suggest potential selection bias, although the direction and impact of such bias are difficult to predict given the aggregate nature of FAERS data (Supplementary Table S1).

As shown in Figure 2, the largest attrition occurred during deduplication and exclusion of non–primary suspect reports. This visual summary highlights how a broad initial FAERS query narrows to a more clinically meaningful set of torsades and QT-prolongation events.

Flow diagram illustrating the extraction and filtering process used to identify QT-related adverse event reports from the FAERS database (January 2015 to October 2025). Reports were restricted to adults (>18 years), primary suspect cases, and MedDRA Preferred Terms for torsades de pointes (PT) and electrocardiogram QT prolonged (PT). Final QT-related event counts were 128 for ondansetron and 245 for olanzapine.

### Narrow-term QT events

#### Ondansetron

Ondansetron had 128 narrow-term QT-related events among 2,608 total reports, producing a reporting rate of 4.91 percent. Disproportionality metrics demonstrated a strong signal, with an ROR of 27.24 (95% CI 22.78–32.58) and a PRR of 25.96 (95% CI 21.90–30.77). The chi-square statistic was markedly elevated at χ^2^ = 3012.31, far exceeding the Evans threshold of 4, indicating that observed counts differed substantially from expected frequencies.

#### Olanzapine

Olanzapine had 245 narrow-term events among 15,257 reports, yielding a reporting rate of 1.61 percent. A disproportionality signal was present but smaller in magnitude compared with ondansetron. The ROR was 8.70 (95% CI 7.65–9.88), and the PRR was 8.58 (95% CI 7.56–9.72), with a chi-square value of χ^2^ = 1594.11, also meeting statistical significance criteria.

Across narrow, high-specificity terms, ondansetron demonstrated a substantially higher relative reporting of QT-related events than olanzapine.

### Broad-term ventricular arrhythmia events (sensitivity analysis)

#### Ondansetron

Using the broader ventricular arrhythmia definition, ondansetron had 217 qualifying events, producing a reporting rate of 8.32 percent. The disproportionality signal remained significant, with an ROR of 13.31 (95% CI 11.58–15.30) and a PRR of 12.28 (95% CI 10.81–13.96). The chi-square statistic was χ^2^ = 2239.14.

#### Olanzapine

Olanzapine had 413 broad-term events, yielding a reporting rate of 2.71. Signal strength decreased compared with the narrow-term definition but remained statistically elevated, with an ROR of 4.09 (95% CI 3.71–4.52), a PRR of 4.01 (95% CI 3.64–4.41), and a chi-square value of χ^2^ = 924.96.

As expected, signal magnitudes were attenuated when broader and less-specific arrhythmia terms were included; however, the overall pattern persisted, with ondansetron consistently demonstrating higher disproportionality than olanzapine (Table 1).

**TABLE 1:** Disproportionality metrics for QT-Related events reported with ondansetron and olanzapine.

Event definition	Drug	Event count (a)	Total PS reports (a+b)	ROR (95% CI)	PRR (95% CI)	Chi-square (χ^2^)
Narrow-term QT events	Ondansetron	128	2,608	27.24 (22.78–32.58)	25.96 (21.90–30.77)	3012.31
​	Olanzapine	245	15,257	8.70 (7.65–9.88)	8.58 (7.56–9.72)	1594.11
Broad-term ventricular arrhythmia events	Ondansetron	217	2,608	13.31 (11.58–15.30)	12.28 (10.81–13.96)	2239.14
​	Olanzapine	413	15,257	4.09 (3.71–4.52)	4.01 (3.64–4.41)	924.96

#### Sensitivity analysis: primary and secondary suspect reports

When the analysis was expanded to include both PS and SS reports, the overall pattern persisted (Supplementary Table S2). For ondansetron, the PS + SS analysis yielded 252 narrow-term QT events among 6,684 total reports, producing a reporting rate of 3.77 percent (ROR 21.46, 95% CI 18.89–24.37; PRR 20.69, 95% CI 18.30–23.39). For olanzapine, the PS + SS analysis identified 411 events among 24,753 reports, with a reporting rate of 1.66 percent (ROR 9.37, 95% CI 8.48–10.35; PRR 9.23, 95% CI 8.37–10.18).

The directional findings remained consistent with the primary analysis, with ondansetron maintaining a substantially higher disproportionality signal than olanzapine across both case definitions. Ondansetron’s ROR decreased modestly from 27.24 to 21.46 (21 percent attenuation), while olanzapine’s ROR showed a slight increase from 8.70 to 9.37. The non-overlapping confidence intervals between drugs persisted in the sensitivity analysis, reinforcing the robustness of the observed difference in QT-related reporting patterns (Supplementary Table S2).

#### Subgroup analyses (narrow-term cohort)

**FIGURE 3:**
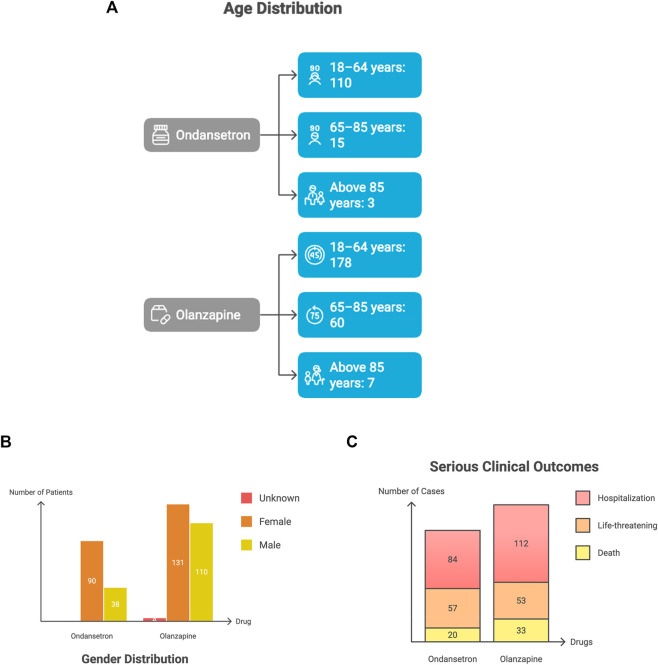
**(A)** Age distribution of narrow-term QT-Related reports. **(B)** Gender distribution. **(C)** Serious clinical outcomes.

Age distributions showed that most QT-related reports for both ondansetron and olanzapine occurred in adults between 18 and 64 years, with progressively fewer cases in the older age groups. Figure 3A demonstrates that most narrow-term QT cases cluster in adults aged 18–64 years, which likely reflects real-world prescribing patterns rather than age-dependent biology.

Age subgroup counts for ondansetron and olanzapine among adults with torsades de pointes or QT prolongation. Most reports were observed in the 18–64 years group, with smaller numbers in the older age categories.

Figure 3B further shows a clear female predominance across both agents, a pattern consistent with established sex-related differences in repolarization reserve.

Gender distribution for QT-related adverse event reports associated with ondansetron and olanzapine. Female patients accounted for most reports across both drugs.

Figure 3C indicates that hospitalization and life-threatening events make up the majority of serious categories, underscoring that most reported arrhythmias occurred in clinically significant settings rather than being incidental ECG findings.

Serious FAERS outcomes for ondansetron and olanzapine among narrow-term QT-related events. Hospitalization was the most frequently reported serious category for both drugs, followed by life-threatening events. Deaths were also reported for each medication.

### Stratified analyses

Temporal Trends: The disproportionality signal for ondansetron remained elevated across all calendar periods, with reporting odds ratios of 23.67 in 2015–2018, 32.64 in 2019–2021, and 42.61 in 2022–October 2025. Similarly, olanzapine demonstrated persistent QT-related signals over time, with RORs of 7.97, 6.88, and 9.32 across the same respective periods (Supplementary Table S3).

Geographic Region: When analyses were restricted to U.S. reports, QT-related disproportionality signals remained statistically significant for both drugs and were directionally consistent with the primary analysis, with ondansetron demonstrating a substantially stronger signal than olanzapine (Supplementary Table S4).

## Discussion

The FAERS patterns suggest that ondansetron and olanzapine generate QT-related safety signals through different pathways and in different clinical circumstances. Ondansetron shows a clearly stronger disproportionality signal, but this becomes meaningful only when viewed in the context of its mechanism, its perioperative use patterns, and the type of clinical environments in which arrhythmias are typically detected. Olanzapine’s signal is smaller in magnitude, yet it consistently appears in situations where vulnerability is already present, which mirrors the conditional risk described in electrophysiologic and post-marketing literature.

### Mechanistic interpretation

Several strands of evidence point in a similar direction regarding ondansetron’s QT-prolonging tendencies. Patch-clamp experiments show that the drug inhibits the hERG potassium channel at submicromolar concentrations, slowing the repolarizing current that keeps ventricular recovery on track. Once that current falters, repolarization drifts, the QT interval lengthens, and early afterdepolarizations become more likely, a prelude to torsades de pointes (Kuryshev et al., 2000).

Translational work has echoed this. In failing rabbit hearts, therapeutic concentrations of ondansetron prolonged action potential duration and QTc, increased early afterdepolarizations, and made the myocardium more vulnerable to malignant rhythms. These observations were linked to both hERG inhibition and effects on small-conductance calcium-activated potassium channels (Yin et al., 2020).

Clinical data follow the same general pattern. Even standard IV doses (four to eight mg) can nudge the QTc upward, and this contributed to the FDA’s decision to withdraw the 32 mg IV dose. NCCN guidance notes the same association, tying ondansetron to QT prolongation and rare torsades cases (Berger et al., 2017).

One recent pharmacovigilance study adds a slightly different nuance: ondansetron tended to show a “random failure” pattern in time-to-event analysis, rather than an early clustered risk (Tan et al., 2024). That irregular timing hints at something clinicians often suspect coexisting factors such as electrolyte shifts, structural disease, or interacting QT-prolonging agents intermittently push a susceptible patient over the threshold.

Olanzapine behaves differently. Its disproportionality signal (ROR ∼8) is not trivial, but it is far lower than ondansetron’s. This fits with clinical trials and antipsychotic comparisons showing only small mean QTc changes (≈1–3 msec) (Aronow and Shamliyan, 2018). Still, the drug is not exempt from concern. Our dataset includes 245 TdP reports in adults, echoing prior post-marketing analyses that identified similar clusters of QT prolongation and nearly a hundred TdP events (Poluzzi et al., 2013; Li et al., 2023). Most cases surfaced in permissive settings of polypharmacy, metabolic derangements, underlying cardiac issues that defined as a conditional risk.

The oncology environment makes this particularly relevant. CINV regimens often combine olanzapine with ondansetron, corticosteroids, NK1(Neurokinin-1 receptor) antagonists, and other agents with their own electrophysiologic effects. A recent prospective trial documented QTc increases over 3 days in women receiving olanzapine, ondansetron, and domperidone, including two patients who exceeded 500 ms (Kamath et al., 2021). Conversely, a few studies have shown slight QT shortening after IM dosing (Aronow and Shamliyan, 2018), a reminder that context matters more than a single mean value. Overall, olanzapine’s electrophysiologic footprint is modest, but not entirely benign once placed into a regimen already thick with QT-active exposures.

### Clinical implications

Ondansetron remains a reliable antiemetic for both chemotherapy-related and postoperative nausea, as reflected in the NCCN guidelines, yet clinicians should be cautious in patients who already carry QT concerns. This includes those with congenital long QT syndrome, existing QT prolongation, or the many patients who accumulate several QT-active medications over the course of treatment. Both the NCCN and the FDA note that the QT effect is dose related and that the 32 mg IV dose was removed after evidence of increased arrhythmia risk. The issue becomes more visible with intravenous use, particularly in postoperative settings, and it tends to intensify when electrolyte abnormalities or other cardiac risk factors are present (Berger et al., 2017; Al-Ramadan et al., 2025).

For these groups, individualized risk assessment matters more than rigid protocols. ECG monitoring is often straightforward to implement and is supported by the American Heart Association as well as recent discussions in The Lancet Oncology. They point out that pre-existing cardiac disease, electrolyte disturbances, and polypharmacy are the elements that tip patients toward instability. There is no single standardized monitoring pathway, but a low threshold for obtaining a baseline ECG and repeating it when clinically reasonable remains the prevailing guidance (Al-Khatib et al., 2003; Fradley et al., 2021; Giraud et al., 2022).

Palonosetron offers an alternative in certain oncology settings. NCCN guidance favors it in selected patients because its QT signal is weaker and, in some regimens, its antiemetic performance equals or exceeds that of ondansetron. Meta-analyses and clinical trials echo this, showing reduced QT liability without loss of efficacy (Berger et al., 2017).

Olanzapine remains a reasonable addition for most adults who lack major cardiac vulnerabilities. Trial data and palliative care experience suggest that typical doses rarely produce clinically meaningful QT prolongation (Kamath et al., 2021). When patients start with an elevated QTc, the NCCN advises practical steps such as lowering the olanzapine dose, adjusting the antiemetic regimen, or correcting electrolytes before treatment proceeds (Berger et al., 2017; Bonar et al., 2023).

### Indication-specific considerations

An important limitation is that olanzapine reports in FAERS predominantly reflect psychiatric use rather than antiemetic use in oncology, whereas ondansetron reports more consistently represent short-term antiemetic exposure. As a result, the two drugs are embedded in different clinical contexts. Psychiatric populations often have a higher burden of metabolic and cardiovascular comorbidity and greater exposure to multiple psychotropic agents with QT liability, while ondansetron is typically administered for brief periods in oncology or perioperative settings where ECG monitoring is more common and may increase detection of QT abnormalities. Differences in exposure duration and monitoring intensity therefore complicate direct comparison of reporting patterns between the two drugs.

Whether olanzapine’s QT risk differs meaningfully between psychiatric and antiemetic indications remains uncertain. Antiemetic dosing is generally lower and of shorter duration than antipsychotic therapy, and limited prospective data suggest only modest QTc effects at low doses. At the same time, the oncology setting introduces additional vulnerabilities, including electrolyte disturbances, treatment-related cardiac stress, and concurrent QT-prolonging supportive medications, which may modify risk in ways not captured in psychiatric cohorts. Because FAERS does not reliably capture indication for use, direct stratification by clinical context was not feasible; exploratory proxy analyses using co-reported chemotherapy agents should therefore be interpreted cautiously.

### Reporter source and data context

The source of adverse event reports is an important consideration when interpreting FAERS-based findings. Using descriptive information from the FAERS Public Dashboard, the majority of reports for both ondansetron and olanzapine that included QT-related adverse events of interest (torsades de pointes and electrocardiogram QT prolongation) were submitted by healthcare professionals, accounting for approximately 85–90 percent of ondansetron reports and over 90 percent of olanzapine reports. This predominance of clinician-reported cases supports the overall clinical credibility of the dataset.

### Understanding comparative risk in the context of FAERS limitations

Clinicians should read comparative safety signals from FAERS with a certain restraint. Disproportionality tells us how often events are reported, not how often they occur, and the distance between those two can be wide because of bias, uneven monitoring, and clinical context.

Importantly, the disproportionality estimates reported in this study are not intended to support formal statistical testing of whether the ondansetron signal exceeds that of olanzapine. Although the lack of overlap between the 95% confidence intervals for the reporting odds ratios (22.78–32.58 for ondansetron versus 7.65–9.88 for olanzapine) is suggestive of a difference in reporting patterns, this finding should be interpreted with appropriate caution. Comparisons across drugs in FAERS are inherently influenced by differences in indication, underlying patient populations, and clinical monitoring intensity, all of which may affect reporting behavior independent of true risk.

FAERS remains useful for detecting early safety patterns, but every strength comes with a limitation. Reporting is voluntary and shifts with external influences. An arrhythmia that appears minutes after intravenous ondansetron in an operating room is almost impossible to miss. The setting is highly monitored, clinicians are already watching ECG tracings, and causality feels temporally intuitive. Episodes linked to chronic olanzapine use in psychiatric care look different. They may be subtle, occur outside continuous monitoring, or be attributed to underlying illness, so the likelihood of a report decreases (Gassman et al., 2017; Fusaroli et al., 2025).

Stimulated reporting complicates things further. FDA alerts or prominent publications can temporarily increase report volume for a specific drug and event. Media coverage can do the same, although the effect is uneven and does not necessarily represent a true shift in clinical risk (Hoffman et al., 2014). Restriction to primary suspect reports can introduce attribution bias, particularly when prior awareness of QT-prolonging effects differs between drugs. Ondansetron has been subject to prominent FDA safety communications, which may increase the likelihood that reporters identify it as the primary suspect when QT events occur, thereby amplifying its apparent signal relative to olanzapine. In sensitivity analyses that included secondary suspect reports, the overall directional pattern remained unchanged, with ondansetron continuing to demonstrate a reporting odds ratio more than twice that of olanzapine (21.46 vs. 9.37). As expected, expansion to a broader case definition led to attenuation of the ondansetron signal (approximately 21 percent), whereas the olanzapine signal showed minimal change, a pattern that may reflect differences in attribution or reporting behavior rather than true risk alone. Importantly, the persistence of non-overlapping confidence intervals across case definitions suggests that the observed contrast is not entirely explained by reporting bias.

Olanzapine’s broad outpatient use adds another layer. Events may be missed, mislabeled, or never submitted. Chronic exposure in patients with multiple comorbidities and complex medication lists dilutes any signal, and the denominator of truly exposed patients is unknown. Without that denominator, incidence is unknowable (Gassman et al., 2017; Raschi et al., 2013; Cepaityte et al., 2021).

Confounding is a constant challenge. Age, illness severity, co-medications, and underlying disease can all distort signals. Patients receiving ondansetron may be postoperative or have cancer, both of which independently increase arrhythmia risk. Patients receiving olanzapine may have psychiatric or metabolic conditions associated with sudden cardiac death (Fusaroli et al., 2025).

Several sources of residual confounding should be acknowledged. Differences in clinical context, including indication, dosing, route of administration, monitoring intensity, and concomitant medication use, may influence both true QT risk and reporting behavior and cannot be fully accounted for within FAERS. In particular, polypharmacy is common in the populations receiving these agents, and concomitant use of other QT-prolonging medications may contribute to observed signals.

In addition, certain stratified or exclusion-based analyses were limited by the available analytic tools. Neither OpenVigil nor the FAERS Public Dashboard provides a reproducible mechanism to systematically identify co-reported medications or stratify disproportionality analyses by reporter type within specific adverse event subsets. As a result, residual confounding related to reporting source and concomitant drug exposure cannot be excluded. These constraints are inherent to spontaneous reporting systems and underscore that the present findings should be interpreted as descriptive and hypothesis-generating rather than causal.

Bayesian shrinkage approaches such as the multi-item Gamma Poisson Shrinker (MGPS) offer a complementary strategy for signal detection, particularly when drug–event counts are sparse. In this study, implementation of MGPS was not feasible because it requires case-level FAERS data and specialized analytic platforms beyond those available. Given the relatively large number of QT-related reports for both drugs, conventional disproportionality metrics (ROR and PRR) were considered appropriate for this hypothesis-driven analysis. MGPS remains a useful approach for future studies, especially those focused on rarer outcomes or smaller subgroups.

For these reasons, FAERS findings should be interpreted with a steady, context-aware hand. Both ondansetron and olanzapine merit thoughtful consideration, and the absolute risk for any individual depends on their comorbidities, medication exposures, and clinical setting. Disproportionality analysis is best viewed as hypothesis generating. Regulatory actions that rely solely on FAERS signals are not always supported by later controlled studies, and many signals fade once examined under more rigorous conditions (Gassman et al., 2017; Fusaroli et al., 2025; Dhodapkar et al., 2022).

### Integration evidence for safer decisions-making

Integrating pharmacovigilance signals with mechanistic and clinical evidence offers a more reliable foundation for evaluating the cardiac safety of ondansetron and olanzapine. FAERS can surface rare events that routine trials are not powered to detect, yet these reports describe patterns of reporting rather than true incidence and are shaped by context, regulatory activity, and even media attention (Dhodapkar et al., 2022). Mechanistic studies help sort signal from coincidence. Ondansetron blocks the rapid delayed rectifier potassium current or hERG channel, which lengthens repolarization in a dose-dependent manner and aligns with the clinical data that prompted removal of the 32 mg IV dose (Kuryshev et al., 2000; Yin et al., 2020; Singh et al., 2023). Olanzapine presents a more conditional pattern, with QT effects emerging mainly when additional vulnerabilities are present, such as electrolyte abnormalities, polypharmacy, or cardiac disease (Kamath et al., 2021; Li et al., 2023; Giraud et al., 2022). These comparative findings should guide questions rather than settle them. Most pharmacovigilance signals need corroboration from prospective or ECG-based clinical studies. In the meantime, several practical steps can help clinicians navigate risk: consider routine QTc monitoring in patients with congenital long QT syndrome, electrolyte derangements, or exposure to multiple QT-active drugs (Al-Khatib et al., 2003; Berger et al., 2017; Freedman et al., 2014); reduce doses when the clinical picture suggests increased susceptibility, particularly with olanzapine (Kamath et al., 2021; Giraud et al., 2022); rethink antiemetic combinations in oncology settings and correct modifiable risk factors before treatment; use alternatives such as palonosetron when QT risk is a priority (Berger et al., 2017; Giraud et al., 2022); and apply targeted ECG and electrolyte checks when IV ondansetron is given in perioperative or emergency care (Li et al., 2018; Orozco et al., 2023). These measures offer a practical way to translate pharmacovigilance insights into safer decision-making at the bedside.

### Future directions

Future work should move beyond spontaneous reporting data to characterize these risks with greater precision. Prospective ECG-based studies in oncology and perioperative populations would help clarify the magnitude and timing of QT changes with each agent. Time-to-onset analyses within electronic health records could further distinguish transient dose-related effects from conditional risk patterns. Oncology-specific risk modeling that incorporates baseline QTc, electrolytes, renal function, and cumulative QT-active drug burden would be particularly valuable. Finally, multicenter registries linking antiemetic dosing, laboratory data, and real-time ECG metrics could provide a more robust denominator and help identify patient groups most likely to benefit from enhanced monitoring or alternative antiemetic strategies.

## Conclusion

Stepping back from the individual numbers, the overall pattern in FAERS is fairly steady. Ondansetron shows a stronger and more consistent QT-related signal than olanzapine, a pattern that fits with its known electrophysiologic and regulatory history. The difference should not be overstated, but it does help clinicians think more carefully about how these drugs behave outside controlled trials, particularly in oncology and perioperative settings.

Olanzapine’s signal is smaller, but not absent. What seems to matter is the environment in which it is used. Many oncology patients receive several QT-active agents in the same week, often amid electrolyte shifts, acute stress, or underlying cardiac disease. In that setting, even a drug with a mild QT footprint can amplify an existing vulnerability. FAERS reports reflect this dynamic in a way that feels clinically recognizable.

These observations should guide, not dictate, bedside decisions. Pharmacovigilance data cannot estimate incidence, yet they remind clinicians that attention to QT-related factors remains worthwhile when assembling multi-agent antiemetic regimens. Correcting electrolytes, reviewing QT-active co-medications, considering baseline ECGs, and using alternatives such as palonosetron for higher-risk patients are all practical steps that align with current guidance.

In short, ondansetron remains a reliable antiemetic but warrants added caution in patients with QT concerns. Olanzapine is generally safe, provided clinicians remain aware of the broader pharmacologic environment. These findings add another small but useful piece to the real-world safety picture and may support more individualized antiemetic planning in oncology, where risk factors often cluster.

## Data Availability

The original contributions presented in the study are included in the article/Supplementary Material, further inquiries can be directed to the corresponding author.
