# Supplementary material for: QT-related adverse events with ondansetron and olanzapine: a real-world FAERS analysis with implications for oncology anti-emetic practice
Source: Front Pharmacol. 2026 Feb 20;17:1748635. doi: 10.3389/fphar.2026.1748635 (PMC12963283; doi:10.3389/fphar.2026.1748635)
Supplement: Supplementary file 1 [file Table1.docx]

Supplementary Table S1: Exclusion Flow and Missingness by Drug

| **Exclusion Criterion** | **Ondansetron n (%)** | **Olanzapine n (%)** |
| --- | --- | --- |
| Initial reports identified | 16,092 (100%) | 49,169 (100%) |
| Excluded: Missing age | 3,755 (23.3%) | 13,620 (27.7%) |
| Excluded: Pediatric age (0–17 years) | 1,328 (8.3%) | 2,554 (5.2%) |
| Excluded: Non–primary suspect designation | 8,401 (52.2%) | 17,738 (36.1%) |
| Final analytic cohort (adult, PS) | 2,608 (16.2%) | 15,257 (31.0%) |

Supplementary Table S1 summarizes the stepwise exclusion process used to derive the final analytic cohorts for ondansetron and olanzapine from the FAERS database during the study period. Report counts and proportions are shown for exclusions due to missing age information, pediatric age (<18 years), and non–primary suspect drug designation. Percentages are calculated relative to the initial number of reports identified for each drug. The final analytic cohort includes adult reports in which the drug of interest was designated as the primary suspect.

|  | Case Definition | Total Reports (a+b) | Narrow-Term QT Events (a) | Reporting Rate (%) | ROR (95% CI) | PRR (95% CI) | Chi-square (χ²) |
| --- | --- | --- | --- | --- | --- | --- | --- |
| Ondansetron | PS only (Primary Analysis) | 2,608 | 128 | 4.91 | 27.24 (22.78–32.58) | 25.96 (21.90–30.77) | 3012.31 |
|  | PS + SS (Sensitivity Analysis) | 6,684 | 252 | 3.77 | 21.46 (18.89–24.37) | 20.69 (18.30–23.39) | 4589.95 |
| Olanzapine | PS only (Primary Analysis) | 15,257 | 245 | 1.61 | 8.70 (7.65–9.88) | 8.58 (7.56–9.72) | 1594.11 |
|  | PS + SS (Sensitivity Analysis) | 24,753 | 411 | 1.66 | 9.37 (8.48–10.35) | 9.23 (8.37–10.18) | 2888.58 |

Supplementary Table S2. Sensitivity Analysis Using Expanded Suspect-Role Definitions

Supplementary Table S2 presents QT-related disproportionality metrics for ondansetron and olanzapine using two case definitions: primary suspect (PS) reports only (primary analysis) and reports in which the drug was listed as either a primary or secondary suspect (PS+SS). Narrow-term QT events include torsades de pointes and electrocardiographic QT prolongation. Reporting odds ratios (RORs), proportional reporting ratios (PRRs), and chi-square statistics are shown to assess the robustness of signal patterns to alternative suspect-role definitions.

Supplementary Table S3. Temporal Stratification of QT-Related Signals by Calendar Period

| **Ondansetron (Primary Suspect)** | **Calendar Period** | **QT Events (a)** | **Total PS Reports (a+b)** | **Reporting Rate (%)** | **ROR (95% CI)** | **PRR (95% CI)** | **Chi-square (χ²)** |
| --- | --- | --- | --- | --- | --- | --- | --- |
|  | 2015–2018 | 41 | 611 | 6.71 | 23.67 (17.22–32.54) | 22.15 (16.46–29.81) | 801.91 |
|  | 2019–2021 | 49 | 580 | 8.45 | 32.64 (24.32–43.81) | 29.97 (22.88–39.24) | 1331.06 |
|  | 2022–Oct 2025 | 41 | 1,538 | 2.67 | 42.61 (31.13–58.33) | 41.50 (30.56–56.35) | 1542.12 |
| **Olanzapine (Primary Suspect)** | **Calendar Period** | **QT Events (a)** | **Total PS Reports (a+b)** | **Reporting Rate (%)** | **ROR (95% CI)** | **PRR (95% CI)** | **Chi-square (χ²)** |
|  | 2015–2018 | 35 | 6,825 | 0.51 | 7.97 (5.70–11.15) | 7.94 (5.68–11.09) | 201.11 |
|  | 2019–2021 | 79 | 4,161 | 1.90 | 6.88 (5.49–8.61) | 6.76 (5.43–8.43) | 376.08 |
|  | 2022–Oct 2025 | 134 | 4,956 | 2.70 | 9.32 (7.83–11.09) | 9.09 (7.67–10.77) | 930.66 |

Supplementary Table S3 shows QT-related disproportionality metrics for ondansetron and olanzapine stratified by calendar period (2015–2018, 2019–2021, and 2022–October 2025). Analyses were restricted to adult reports in which the drug of interest was designated as the primary suspect. This stratification was performed to evaluate temporal stability of QT-related signals and to assess whether observed patterns persisted across different reporting periods.

Supplementary Table S4. QT-Related Disproportionality Signals Restricted to U.S. Reports

| **Drug** | **QT Events (a)** | **Total PS Reports (a+b)** | **Reporting Rate (%)** | **ROR (95% CI)** | **PRR (95% CI)** | **Chi-square (χ²)** |
| --- | --- | --- | --- | --- | --- | --- |
| Ondansetron (U.S.) | 79 | 913 | 8.65 | 104.57 (82.81–132.06) | 95.61 (77.22–118.38) | 7128.13 |
| Olanzapine (U.S.) | 34 | 3,219 | 1.06 | 11.61 (8.27–16.31) | 11.50 (8.22–16.09) | 312.39 |

Supplementary Table S4 presents QT-related disproportionality metrics for ondansetron and olanzapine restricted to reports originating from the United States. Analyses were limited to adult primary suspect reports and narrow-term QT events. This geographic subanalysis was conducted to assess the robustness of findings within a more homogeneous regulatory and reporting environment and to compare results descriptively with the primary global analysis.

**Abbreviations:** FAERS, FDA Adverse Event Reporting System; PS, primary suspect; SS, secondary suspect; QT, QT prolongation; ROR, reporting odds ratio; PRR, proportional reporting ratio.
